# Electronic and Magnetic Properties of Lanthanum and Strontium Doped Bismuth Ferrite: A First-Principles Study

**DOI:** 10.1038/s41598-018-37339-3

**Published:** 2019-01-17

**Authors:** Ayana Ghosh, Dennis P. Trujillo, Hongchul Choi, S. M. Nakhmanson, S. Pamir Alpay, Jian-Xin Zhu

**Affiliations:** 1 Department of Materials Science & Engineering and Institute of Materials Science, University of Connecticut, Storrs, CT 06269 USA; 2 Theoretical Division, Los Alamos National Laboratory, Los Alamos, NM 87545 USA; 3 Department of Physics, University of Connecticut, Storrs, CT 06269 USA; 4 Center for Integrated Nanotechnologies, Los Alamos National Laboratory, Los Alamos, NM 87545 USA

## Abstract

While bismuth ferrite BiFeO_3_ (BFO) is a well studied multiferroic material, its electronic and magnetic properties in the presence of A-site dopants have not been explored widely. Here we report the results of a systematic study of the local electronic structure, spontaneous polarization, and magnetic properties of lanthanum (La) and strontium (Sr) doped rhombohedral bismuth ferrite within density functional theory. An enhanced ferroelectric polarization of 122.43 *μ*C/cm^2^ is predicted in the uniformly doped BiLaFe_2_O_6_. We find that substitution of Sr in the A-site drives the system into a metallic state. The nature of magnetism arises mainly from the B-site Fe exhibiting a G-type antiferromagnetic ordering. Our study finds that upon dopant substitution, the local magnetic moment is decreased and its magnitude is dependent on the distance between the Fe and the dopant atom. The correlation between the local moment and the distance between the Fe and the dopant atom is discussed.

## Introduction

The correlation between magnetic, electronic, and ferroelectric properties of transition-metal oxides has been studied for several technological applications ranging from tunable multifunctional spintronics to magnetoelectric random access memory devices and various kinds of optoelectronic devices. The most well-studied single-phase multiferroic^1–13^ material is the perovskite BiFeO_3_ (BFO)^3^, which shows a strong coupling between the spontaneous electric polarization and magnetic ground state. BFO undergoes two phase transitions upon cooling; one at its Curie temperature of 830 °C, from a paraelectric to ferroelectric and one where it becomes magnetically ordered at its Néel temperature of 370 °C. Hence, the low temperature phase with rhombohedrally distorted perovskite structure, which is both ferroelectric and anti-ferromagnetic, is of interest to our research. This phase has been reported both theoretically and experimentally, to show high spontaneous polarization and weak ferromagnetic ordering. The large orbital radius of the Bi 6*s*^2^ lone pairs is responsible for the spontaneous electric polarization whereas the magnetism originates from Fe 3*d* electrons. One of the challenges is that BFO thin films exhibit low electrical resistivity^14^, which limits its application in designing novel multifunctional non-volatile random-access memory devices.

Recent research to enhance electronic and magnetic properties of BFO has shown that the presence of common A-site dopants such as La and Sr can have a significant effect on decreasing leakage current in BFO thin films^15–23^. Furthermore, such additions may also control the volatile nature of Bi atoms, whereas B-site dopants are also studied in order to enhance the magnetic ordering in BiFeO_3_ which may help with loss and leakage^24–36^. Formation of oxygen vacancies may also be suppressed in the presence of these dopants due to charge compensation. Oxygen vacancies have also been shown to affect significantly the electronic and magnetic properties of related double perovskites^37^. Therefore, improving the antiferromagnetic ordering along with retaining high spontaneous polarization of BFO through judicious doping to make it technologically more feasible has captured much interest in the condensed matter and materials physics communities. We note that both La and Sr are commonly used dopants in prototypical ferroelectrics such as PLZT ((Pb_1−*x*_La_*x*_)(Zr_1−*y*_Ti_*y*_)O_3_), SBT (SrBi_2_(Ta_2_O_9_)), SBN (SrBi_2_(Nb_2_O_9_)), BST (Ba_*x*_Sr_1−*x*_TiO_3_) and BIT (Bi_4_Ti_3_O_12_) to improve characteristic functional/physical properties. In lead zirconate titanate (PZT) thin films, La^3+^ dopants help to increase dielectric constant and reduce the coercive field to make it more useful for ferroelectric memory and sensing/actuating applications^38,39^. For BIT thin films, La^3+^ helps to improve fatigue characteristics while maintaining high remanent polarization^40,41^. Sr^2+^ in BST thin films shifts its paraelectric-ferroelectric transformation temperature to near room temperature for applications in telecommunications which require highly electrically tunable dielectric responses^42^. The presence of Bi^3+^ improves dielectric properties in SBT and SBN thin films by increasing its Curie temperature^43^. Previous reports^44,45^ also indicate suppression of local magnetic moments and enhancement of overall magnetic ordering in the presence of Sr doping while electronic properties and size of ferroelectric polarization for La-doped BFO is comparable^46–48^ to that of pristine BFO in thin films.

The purpose of the present work is to investigate dopant effects, specifically that of La and Sr on the electronic, magnetic and ferroelectric properties of BFO within density functional theory. The paper is organized as follows: Sec. II briefly reviews the computational details of formulating structural optimization, calculations of magnetic moment and spontaneous polarization using the Berry phase method of various supercells within first-principles theory. In Secs. III and IV, we discuss the results of our calculations, progressing from smallest unit cells to bigger supercells. Finally, Sec. V presents concluding remarks.

## Computational Details

We perform first-principles calculations using the projector-augmented plane-wave (PAW) method as implemented in the Vienna *ab initio* Simulation Package^49,50^. The generalized gradient approximation (GGA) is adopted for the exchange-correlation functional. The GGA + U method is introduced to capture the strong correlation in *d* localized orbitals.

We use a Hubbard parameter U_*eff*_ = 2 eV, which is shown to be reasonable in previous calculations^51,52^. We note that other values of U_*eff*_ around 4 eV have also been used in the literature. We have systematically studied the band gap for U_*eff*_ = 0, 2, 4 and 6 eV with the GGA exchange-correlation functional for the pristine BFO in primitive unit cell and obtained values of 1.0 eV, 2.2 eV, 2.5 eV and 3.2 eV respectively. Throughout the work, we report the results for U_*eff*_ = 2 eV. Although the corresponding band gap value is slightly lower than the experimental band gap^53–57^, this difference should not significantly affect the trend in electronic and magnetic properties for doped BFO systems.

For all calculations, we consider G-type antiferromagnetic ordering on Fe cations with spins aligned oppositely in two sublattices.

**Figure 1:**
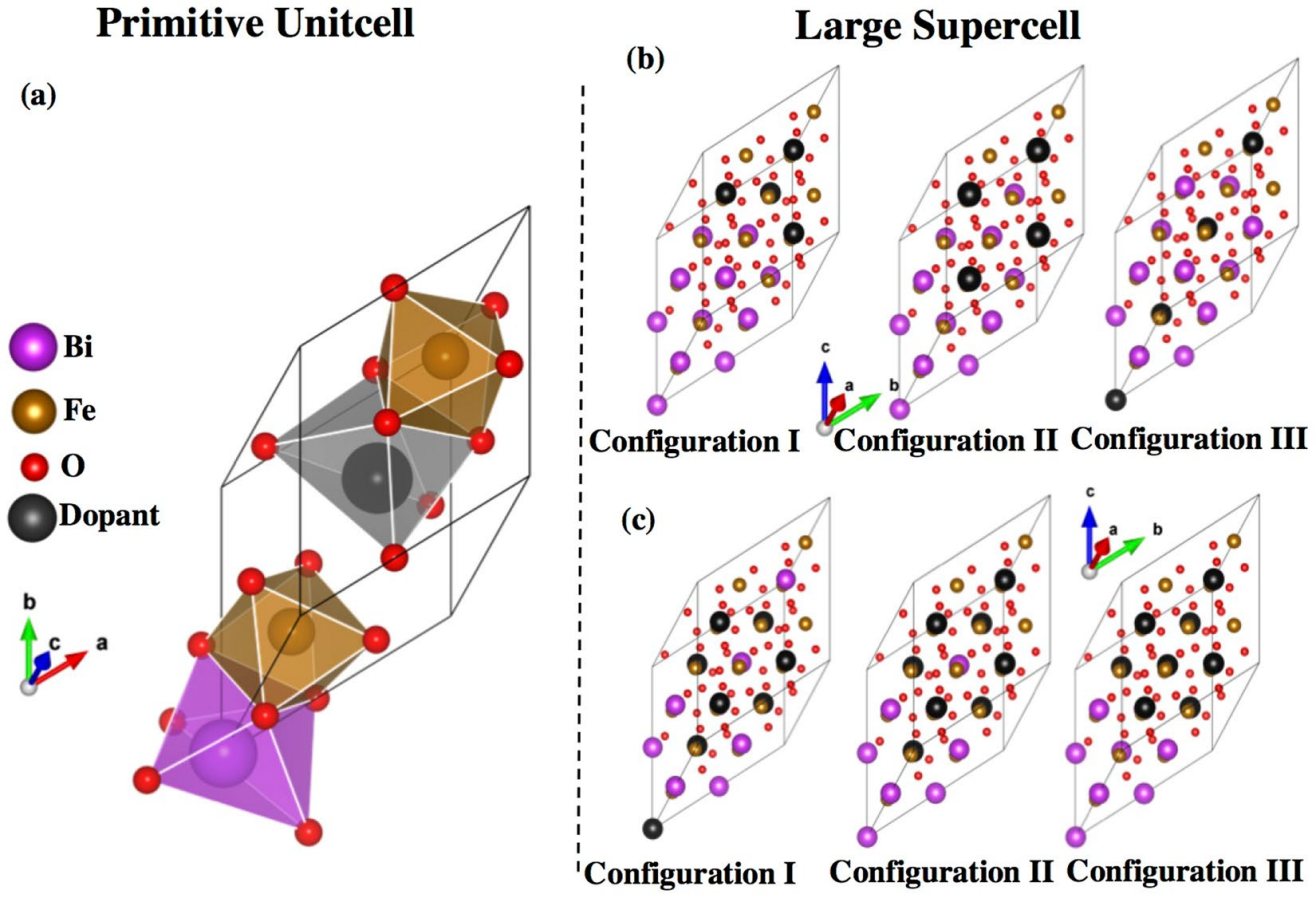
Structural models for (**a**) BiLaFe_2_O_6_ and BiSrFe_2_O_6_. (**b**) 80-atoms unit cell with *x* = 25% and (**c**) *x* = 50% doping in three distinct configurations where distances between dopants are varied.

We first perform the structure optimization of the rhombohedral 2 formula units of (10 atoms) BiFeO_3_ by relaxing the atoms steadily toward the equilibrium until the Hellman-Feynman forces are less than 10^−3^ eV/Å. We refer to this 2 formula unit crystal cell as the primitive unit cell hereafter. We use these lattice parameters to construct the supercells for the exploration of the dopant effects with varying concentrations. In this primitive unit cell, substitution of one La or Sr for one Bi gives rise to compound BiLaFe_2_O_6_ and BiSrFe_2_O_6_ as shown in Fig. 1. These two specific structures also provide us an opportunity to compare the results for 50% doped BFO as calculated in the supercell technique. We note that for the rare-earth-based ferrites the crystal structure can be orthorhombic^58,59^, which could originate from the 4*f*-electron physics. Therefore, we also check the possible structure change for 50% La-doped BFO when we do the ferroelectric polarization calculations and find the *R*3-rhombohedral structure still sustained. The reason lies in the fact that the La has no active 4*f*-electron physics involved.

In the 2 × 2 × 2 supercell with 16 formula units of BFO (80 atoms), we vary the concentration of dopant atoms by 6%, 25% and 50% to study the response of electronic and magnetic properties. For 50% doping, it is also interesting to explore the connection of the results in primitive unit cells and supercells. All calculations for the primitive unit cells and supercells are performed with a 5 × 5 × 5 Monkhorst-Pack^60^ **k**-point mesh^51^ centered at Γ and a 500 eV plane-wave cut off energy, for which the results are converged. For both 25% and 50% concentrations, we build three supercells. For each of them, the distances between the dopant atoms are varied as shown in Fig. 1. For both 25% and 50% dopant concentrations, Configuration III represents the structural model where the dopants are placed at the most elongated position along [111] direction in the supercell. Configuration I has the dopants most packed with respect to each other, i.e. has the minimum average distance between dopants. Configuration II is such designed where the dopant atoms are placed in between these two extremums. We also provide an example of how this average distance between dopants are calculated as well as atomic coordinates of A-site and B-site atoms for all of these configurations in Supplementary Material (See **SM-1** and **SM-2**, respectively). The structural relaxations were performed only for the pristine BFO, 6% doped, 25% and 50% doped in Configuration III, for which the spontaneous polarization exists. We choose Configuration III only because we anticipate that this quantity is insensitive to the distribution of the dopants. These relaxations followed the same convergence criterion as explained previously in the section. The choices of supercells are based on experimental evidence reported elsewhere^15–36^. These particular cases are chosen to explore the location (with respect to Fe atoms) dependence of dopant effects in these supercells.

The macroscopic polarization in an infinite periodic solid can be written as the sum of ionic and electronic terms:

1 $${\boldsymbol{P}}={{\boldsymbol{P}}}_{{\rm{ion}}}+{{\boldsymbol{P}}}_{{\rm{el}}}=\frac{e}{V}\sum _{\tau }{Z}_{\tau }{{\boldsymbol{b}}}_{\tau }-\frac{1}{V}{\int }_{V}{\boldsymbol{r}}\,\rho ({\boldsymbol{r}})\,d{\boldsymbol{r}},$$

where *V* is the volume of the unit cell, *Z*_*τ*_ and ***b***_*τ*_ are the charge and position of the *τ*-th atom in the cell, and *ρ*(***r***) is the cell-periodic density of electron charge. The electron-charge density can be expanded into the sum of occupied Bloch states *ψ*_*i****k****σ*_(***r***) or Wannier functions *W*_*iσ*_(***r***) as represented below within modern theory of polarization^61,62^:

2 $$\rho ({\boldsymbol{r}})=-\,\frac{e}{{\mathrm{(2}\pi )}^{3}}\sum _{i,\sigma \,{\rm{occ}}}{\int }_{BZ}d{\boldsymbol{k}}|{\psi }_{i{\boldsymbol{k}}\sigma }({\boldsymbol{r}}{)|}^{2}=-\,e\sum _{i,\sigma \,{\rm{occ}}}|{W}_{i\sigma }({\boldsymbol{r}}{)|}^{2}.$$

Utilizing the Bloch-state representation for *ρ*(***r***) one can calculate the total electronic contribution of polarization as the solutions to the delocalized Bloch functions along a density-sampled string of neighboring points in **k**-space for a given crystalline geometry. For a specific choice of basis vectors, the ionic contribution is calculated by summing the product of the position of each ion in the unit cell with the nominal charge of its rigid core. Therefore, the total spontaneous polarization is obtained by summing the electronic and ionic part of the polarizations using Berry phase method. Using the Berry phase approach, the total polarization can only be reported up to integer multiples of the indeterminate polarization quanta with the latter computed by e**R**/Ω. Structurally, displacements of ions along [111] and counter-rotations about the oxygen octahedra about [111] of cubic perovskite (undistorted structure) yields rhombohedral *R*3*c* (fully distorted) structures for pristine BFO and *R*3 for La-doped BFO. To avoid any discrepancy in choosing one specific path that connects the smallest polarization of the undistorted phase to that of the fully distorted phase, we only look at the residual polarizations by following the procedure proposed in ref.^51^. In other words, we first calculate the total polarizations of both phases for each system. We then compare those to the polarization quanta and take the difference of their residues for the true polarization.

## Results

In this section, we report comparative results of the electronic, magnetic properties of BiLaFe_2_O_6_ and BiSrFe_2_O_6_ and other doped BFO treated in the supercells.

### BiLaFe_2_O_6_ and BiSrFe_2_O_6_

**Figure 2:**
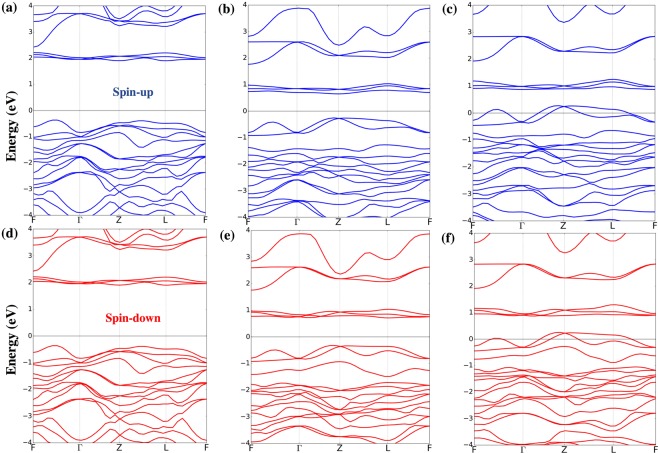
Band structures of (**a**,**d**) pristine BFO, (**b**,**e**) BiLaFe_2_O_6_ and (**c**,**f**) BiSrFe_2_O_6_ in [−4, 4] eV energy range as calculated along high-symmetry-**k**-points for a rhombohedral crystal system along (*F*-Γ-*Z*-*L*-*F*). Blue represents spin-up and red represents spin-down bands. Energy is measured with respect to the Fermi energy.

**Figure 3:**
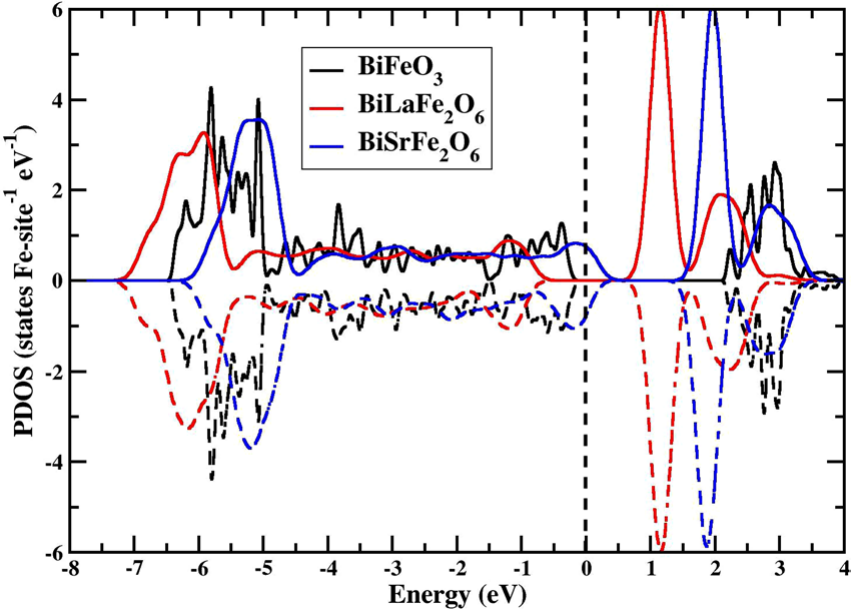
Partial spin-resolved density (spin-up - solid and spin-down - dashed lines) of states of Fe-3d for pristine BFO, BiLaFe_2_O_6_ and BiSrFe_2_O_6_. Energy is measured with respect to the Fermi energy.

We observe the change in the electronic and magnetic properties for BiLaFe_2_O_6_ and BiSrFe_2_O_6_ as compared to pristine BFO. The relaxed structure parameters of the 10-atom unit cell of BFO belonging to *R*3*c* 161 space group are *a* = *b* = *c* = 5.69Å and *α*_*rh*_ = 58.54°. The relaxed cell volume is 125.72 Å^3^. This is in good agreement with previous experimental and theoretical predictions^51,63^. We have used these same lattice parameters for the doped unit cells, noting that the minimal changes in atomic positions during additional structural optimization do not affect the metallic or insulating states obtained for these configurations. The band structure calculations for primitive unit cells are performed along high-symmetry-**k**-points for a rhombohedral crystal system (*F*-Γ-*Z*-*L*-*F*) as shown in Fig. 2. The system remains insulating for pristine BFO and BiLaFe_2_O_6_. The indirect band gap is smaller in BiLaFe_2_O_6_ as compared to BFO. The valence band maxima (VBM) is located in between F and Γ points for BFO while the conduction band minimum (CBM) is located at Z. For BiLaFe_2_O_6_ and BiSrFe_2_O_6_, the VBM and CBM are both located at point Z. The locations of the energy levels as shown in Fig. 2 are referenced with respect to the low-lying O 2*s* states, whose energy is considered to be the same in all the compounds considered in this study. This is done specifically to emphasize the upward/downward shifts of the bands in energy in the doped BiFeO_3_ compounds. A few electronic bands in the valence band (Γ-*Z*-*L*) region are shifted above the Fermi level for BiSrFe_2_O_6_ leading to a metallic state. This is due to the hole doping effect when Sr atoms are substituted for Bi atoms with Sr giving up nominally two electrons in its divalent state. It leads to the shift of the chemical potential with the minor change of bonding nature. As shown in Fig. 3, this corresponds to the crossing of the Fe-3*d* band with the Fermi energy, driving the system to be metallic. Our finding for Sr-doped BFO is consistent with the observation from the electronic structure calculations for SrFeO_3_ in AFM state^64,65^.

The nature of magnetism is assumed to be G-type^66–68^ arising from Fe atoms. In addition, with the A-site substitution, the results from our first-principles calculations follow the Goodenough-Kanamori rules that the partially filled d orbitals of two Fe ions can have a strong antiferromagnetic superexchange coupling when they form a 180 degree Fe^3+^-O^2−^-Fe^3+^ bonds. A table listing total energies for all doped and undoped systems in the primitive unit cell and the supercell with G-type AFM and FM ordering is given in Supplementary Material (See **SM-3**). For the pristine BFO, the average of the staggered magnetic moment is 4.02 *μ*_*B*_ per Fe, which reduces to 3.95 *μ*_*B*_ and 3.74 *μ*_*B*_ for La and Sr doped BFO, respectively. Therefore, A-site dopants will overall suppress the magnetic moments in these systems. More importantly, the net magnetic moment for pristine BFO is 0 *μ*_*B*_ whereas net moments for the La, Sr cases are 0.004 *μ*_*B*_ and −0.630 *μ*_*B*_, respectively.

Since calculations of spontaneous polarization demand the system to be perfectly insulating, we cannot report any polarization for Sr doped BFO. For the pristine BFO and 50% La doped BFO, the values of the polarization are 114.90 *μ*C/cm^2^ and 122.43 *μ*C/cm^2^ respectively. These are the largest reported polarizations of these systems in these specific geometric configurations. The change in polarization of *R*3 BiLaFe_2_O_6_ as compared to *R*3*c* pristine BiFeO_3_ is due to difference in ionic radii and local charge distribution of these A-site cations. For Bi (5*d*^10^ 6*s*^2^ 6*p*^3^) there are total 3 valence electrons whereas 3 for La (5*s*^2^ 5*p*^6^ 5*d*^1^ 6*s*^2^) and 2 for Sr (4*s*^2^ 4*p*^6^ 5*s*^2^). For pristine BFO, there is a hybridization between 6s and 6p states that gives rise to active lone pair electrons. For La and Sr doped cases, the degree of Fe 3*d*-4*p* orbital mixing^23^ is reduced. This is due to the movements of local ions to a more symmetric position. This is further analyzed by Bader charge density approach^69–72^ as reported in Sec. III.C where the local charge distributions are compared for La and Sr doped BFO to that of pristine BFO. Because of these, the ionic and electronic contributions to the total polarization for La doped BFO are higher than those of the pristine BFO, leading to a higher spontaneous polarization.

### Supercell

**Figure 4:**
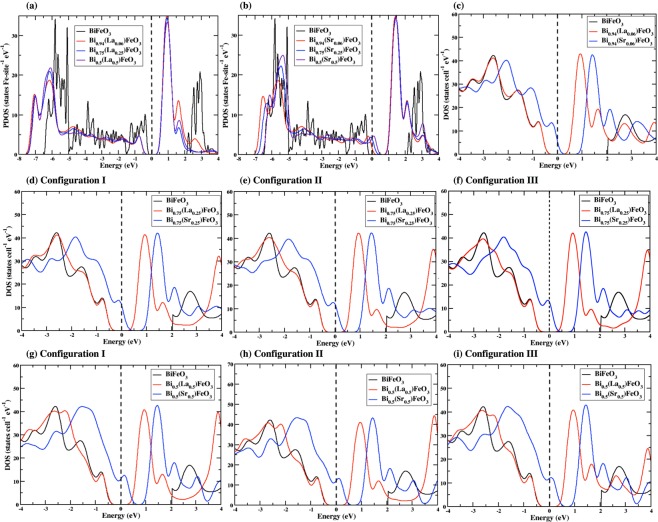
Partial density of states of Fe-3d orbitals on atoms closest to the (**a**) La and (**b**) Sr atoms for 6%, 25% and 50% (Configuration III) concentrations respectively. Total electron density of states for 6% doped (**c**), 25% doped (**d**–**f**) and 50% doped (**g**–**i**) BFO in all three configurations. Energy is measured with respect to the Fermi energy.

To observe and quantify the effects of dopants more rigorously, we have constructed 80-atom supercells with *a* = *b* = *c* = 11.37Å and *α*_*rh*_ = 58.54 as shown in Fig. 1. The total electron density of states for both spin-resolved channels have been computed for 6%, 25%, and 50% La, Sr doped BFO. The results are plotted in Fig. 4 within specific energy ranges in comparison to that of the pristine BFO. Figure 4(b) shows that by introducing only 6% Sr doping, which amounts to only one Sr atom in the BFO supercell, the system goes to a metallic state^73^; while the La-doped BFO remains insulating. This behavior also suggests a *p*-type electrical behavior in Sr-doped BFO, where holes are the main charge carriers. As the dopant concentration is increased from 6% to 25% and 50%, these electronic properties are more pronounced as shown in Fig. 4(c–h). Even for 50% dopant concentration, La doped BFO maintains an insulating state.

For 6% dopant concentration, the Fe atom with the smallest distance 3.30 Å away from the dopant has the local magnetic moments of 4.03 *μ*_*B*_ for La and 3.98 *μ*_*B*_ for Sr cases, respectively; while the Fe atom with distance 3.11 Å away from the dopant has the local moment of −4.03 *μ*_*B*_ for La and −3.96 *μ*_*B*_ for Sr cases, respectively. In addition, the local moment on the other Fe atoms varies with their distance from the dopant site, suggesting a strong dependency on the atomic environment. The Fe atom with the farthest distance from the dopant has the largest local magnetic moments. We report local moments for each Fe atom along with its distance from the dopant atoms for both La and Sr cases in Supplementary Material (See **SM-4**). As the distance between the dopants to Fe atoms increases, the doped case behaves more like the pristine and the effects of the dopants are less pronounced. Similar behavior has been obtained for 25% and 50% dopant concentrations and the results are summarized in Table I. Overall, A-site dopants suppress magnetic moments. In our calculations, we obtained average magnetic moment of 4.04 *μ*_*B*_ (4.00) for 6% La (Sr) concentrations. For 25% La (Sr) concentrations, the average magnetic moments are 4.03 *μ*_*B*_ (3.78), 4.04 *μ*_*B*_ (3.88) and 4.03 *μ*_*B*_ (3.88) in Configuration I, II and III, respectively. For 50% La (Sr) concentrations, the average magnetic moments are 4.03 *μ*_*B*_ (3.75), 4.03 *μ*_*B*_ (3.76) and 4.03 *μ*_*B*_ (3.75) in Configuration I, II and III respectively. We remark that our results for 50% concentration in the Configuration III supercell calculations are consistent with those in primitive unit cells.

Overall, the difference in the highest and the lowest local moment for 6%, 25% and 50% dopant concentrations are listed in Table 1. The overall magnetic moment of Fe atoms is decreased as the dopant concentration is increased. The change is the greatest when BFO is doped with 50% Sr which suggests that the Sr dopant has a greater impact on the magnetic properties compared to La because of the dissimilar nature of its A-site cations as compared to pristine BFO.

**Table 1:** Maximum and minimum local magnetic moments (*μ*_*B*_) of Fe in the doped BFO with varying concentrations.

	La (Max)	La (Min)	Sr (Max)	Sr (Min)
(1) 6%	4.04	4.03	4.02	3.98
(2) Configuration I: 25%	4.05	4.03	3.98	3.77
(3) Configuration II: 25%	4.04	4.03	3.95	3.85
(4) Configuration III: 25%	4.04	4.03	3.90	3.88
(5) Configuration I: 50%	4.04	4.02	3.88	3.63
(6) Configuration II: 50%	4.04	4.02	3.88	3.71
(7) Configuration III: 50%	4.04	4.03	3.77	3.73

We also report the polarizations (**P**) of pristine, 6%, 25%, 50% La-doped BFO as 115.61 *μ*C/cm^2^, 116.59 *μ*C/cm^2^, 118.71 *μ*C/cm^2^ and 120.09 *μ*C/cm^2^, respectively. There is a 7–10% change in polarization as is shown in Table 2. Again our results on the polarization for the 50% La-doped case in Configuration III is consistent with that reported with that reported for the primitive unit cell. We note that for the pristine BFO, the 0.6% difference in the polarization between the calculations with primitive unit cell and supercell could be attributed to the slight difference in the atomic positions during structural relaxation.

**Table 2:** Spontaneous polarization (*μ*C/cm^2^) of La-doped BFO for various percentages of La doping.

	P	ΔP
(1) Pristine	115.61	0
(2) 6%	116.59	0.98
(3) Configuration III: 25%	118.71	3.10
(4) Configuration III: 50%	120.09	4.48

The relaxed cell volumes corresponding to each case are 1005.78 Å^3^ (pristine), 1023.58 Å^3^ (6% La-doped), 1010.38 Å^3^ (25% La-doped in Configuration III) and 1009.54 Å^3^ (50% La-doped in Configuration III), respectively which is used to calculate the true polarization. It is important to mention that these relaxed supercells still belong to the same rhombohedral *R*3 symmetry group family and thus the reference states for each of these cases are their corresponding cubic perovskites (undistorted) structures with equal volumes of the respective undistorted ones. Furthermore these computations are only performed for one specific structural configuration such as Configuration III since we do not expect major differences in this trend of enhanced spontaneous polarization as the distances between dopants sites are varied. There is no spontaneous polarization for Sr-doped BFO because they are metallic.

## Discussion

From the previous sections, it is evident that the electronic properties of La doped BFO in each supercell is comparable to that of pristine BFO. The differences as shown in the comparative total electron density states in Fig. 4(c–h) arise due to reduction in anisotropy in Fe-O bond distances and local structural distortions as the dopants concentrations are increased. Moreover, for La-doped cases, the Fe 3*d*-4*p* orbital mixing and differences in electronegativities are the additional factors to enhance the spontaneous polarization and magnetic properties. Similarly, for the Sr case, the Fe ion in the FeO_6_ moves to a more symmetric position that gives rise to variation in electron densities. However, the electronegativity of Sr is quite low compared to Bi and the Fe-3*d* band upshift across the Fermi energy makes it non-insulating.

**Figure 5:**
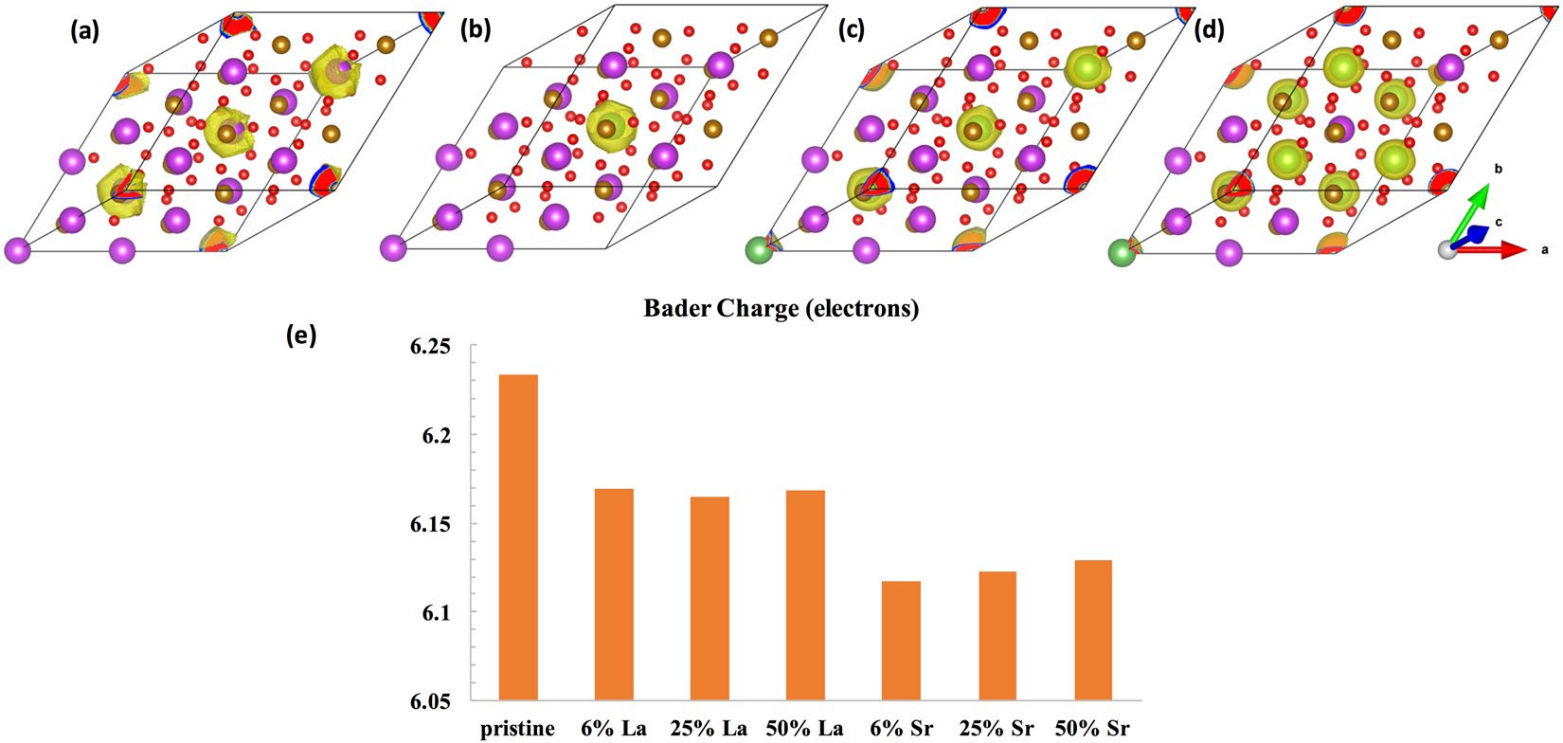
Representative Bader charge volumes of comparative A-site cations in (**a**) pristine and A-site dopants in (**b**–**d**) 6%, 25%, 50% doped BFO corresponding to Configuration III respectively. (**e**) Bader charges of Fe atoms closest to dopant atom in each case.

Here, we also provide a Bader charge analysis in Fig. 5 for pristine BFO as compared to the various doped cases. Bader analysis gives the electronic charge and density of atoms suitable to discuss electronic structure and ionic-covalent character of a compound. The normalization of volume as considered by this technique is necessary to highlight electronic structure modifications due to exact exchange.

The number of valence electrons included in each atomic species pseudo potential are 15e for Bi (5*d*^10^ 6*s*^2^ 6*p*^3^), 8e for Fe (3*d*^7^ 4*s*^1^), 6e for O (2*s*^2^ 2*p*^4^), 11e for La (5*s*^2^ 5*p*^6^ 5*d*^1^ 6*s*^2^) and 10e for Sr (4*s*^2^ 4*p*^6^ 5*s*^2^). We only consider the valence electrons in this analysis since the effects of core electrons on the electronic behavior^74^ of these systems are expected to be minimal. Only one type of configuration (Configuration III) for 25% and 50% is considered here since the overall properties do not vary significantly for different configurations. The Bader charge volumes as represented in Fig. 5(a–d) are suggestive of more symmetric charge distributions around the A-site dopants in BFO, as described earlier. The average Bader charge for Bi cations is 12.32 electrons and 8.92 and 8.42 electrons for La and Sr cations, respectively. Therefore, the estimated valence charges of Bi, La and Sr are 2.68, 2.08 and 1.58 (cf. formal valence charges of 3, 3 and 2), respectively. Figure 5(e) shows the Bader charges of Fe atoms closest to the dopant atoms for varying concentrations. It also implies that valence charge of Fe is close to 2 + (8e −∼ 6e) for pristine and doped BFO compounds. Charges on oxygen atoms are also reduced to ∼1.4- (cf. formal valence charge of 2-) states for all systems to maintain charge neutrality. There is nominal difference in Fe valence states as compared between pristine and La-doped BFO supercells. There is overall ∼2% reduction in the local charges of Fe atoms between pristine and Sr doped BFO, which provides an explanation for the overall reduced moment in this case. Bader charges and volumes (Å^3^) for all the atoms for pristine, 6%, 25% and 50% La and Sr doped BFO (in Configuration III) are also given in Supplementary Material (See **SM-5**, **SM-6** and **SM-7** respectively). We observe the valence charge of the La dopant atoms is higher than that of Sr by 0.5 electrons. This analysis is also suggestive of mixed ionic-covalent character as observed in such compounds^74^.

**Figure 6:**
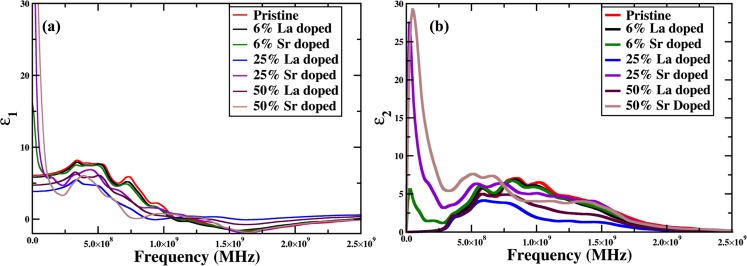
(**a**) Real and (**b**) imaginary part of dielectric functions, *ε*(*ω*) for pristine and 6%, 25% and 50% La, Sr doped BFO supercells.

Finally, we present the optical properties such as dielectric function as a function of frequency and refractive indices to provide a sufficient insight to how La and Sr can improve the energy loss. This computation was performed using Kramers-Kronig relation as implemented in VASP^49,50^. From Fig. 6(a,b), it is clear that both the phase lag and energy loss are improved as dopants are introduced in pristine BFO^75,76^. The dopant sites may no longer be considered as isolated sites in the presence of higher dopant concentrations, which may lead to a dielectric breakdown of such materials when subjected to intense electric fields. From the imaginary part of the dielectric functions, the transitions between O-*p* electron to unoccupied Fe-*d* states, O-*p* electron to *p* states high energy conduction bands and other inner electron excitations can also be identified in each case. The highest value of refractive index n for pristine BFO is reported as 2.88 at an energy of 2.22 eV. For 6% La and Sr doped cases these values shift to 2.82 at an energy of 1.4 eV and 2.84 at 2.12 eV. As the dopant concentration is increased to 25% and 50%, these values decrease to 2.34 and 2.67 at same energies, suggesting the same behavior of major absorption due to transition from valence to conduction bands as predicted by the frequency dependent dielectric functions.

## Concluding Remarks

In conclusion, we have investigated systematically the La and Sr dopant effect on electronic and magnetic properties in BFO. We have found that La-doped BFO maintains an insulating state whereas adding Sr drives the system to be metallic. This behavior is evident from the band structure calculations. We have also obtained an enhancement of the total polarization in La-doped BFO by 4–8% over the pristine BFO, because of the variance in ionic radii, electronegativity and valence electrons in Bi^3+^ and La^3+^. The average magnetic moment of Fe atoms decreases as we increase dopant concentration. This behavior is also dependent on the local atomic environment. In the structural configuration where the distances between dopant atoms with respect to Fe atoms is the smallest, the variance is the highest for lowest local moments. The local charge distribution is responsible for these changes in electronic and magnetic properties. Variance in *s* and *d* orbitals occupancy for La and Sr and *d* orbital occupancy for the Fe-like transition metal ion may also contribute to changes in these properties.

The following remarks are in order: Our study gives an estimate of the magnetic moments in primitive unit cells and supercells as compared to pristine BFO. We note that we do not perform any structural relaxation for *every* supercell because the minimal changes in atomic positions will not affect the metallic or insulating states obtained for the doped system. We anticipate no substantial change in the electronic properties (density of states, band structures) if the impurity concentration is varied in La doped case. Structural relaxations were performed *only* for pristine BFO, 6%, 25% and 50% La doped BFO in Configuration III, for which the spontaneous polarization occurs. The interference caused by large concentration of dopants in BFO does not affect the polarization significantly. The net ferromagnetic component of La and Sr doped BFO is increased as compared to the pristine BFO. This increase gives the potential of ferromagnetic, ferroelectric coupling in at least La-doped BFO. The specific three structural configurations were chosen judiciously to quantify the effects of dopants with respect to wide change in the surrounding atomic environment. We reason that the use of the sample averaging technique^77,78^ for a large number of dopant atoms situated at every possible site may give us deeper insights into these effects. But such calculations will become extremely expensive for relatively large impurity configurations and are therefore beyond the scope of this paper. In addition, we expect that the electronic properties explored here in seven distinct structural configurations with 6%, 25% and 50% concentrations may not differ much compared to other possible structural configurations to cause significant variations in our predictions.

## Supplementary information

Supplementary Materials
