# Supplementary material for: Electronic and Magnetic Properties of Lanthanum and Strontium Doped Bismuth Ferrite: A First-Principles Study
Source: Sci Rep. 2019 Jan 17;9:194. doi: 10.1038/s41598-018-37339-3 (PMC6336767; doi:10.1038/s41598-018-37339-3)
Supplement: Supplementary file 1 — Supplementary Materials [file 41598_2018_37339_MOESM1_ESM.pdf]

Electronic and Magnetic Properties of Lanthanum and Strontium Doped Bismuth Ferrite: A First-Principles Study

Ayana Ghosh,[1,2] Dennis P. Trujillo,[1,2] Hongchul Choi,[2] S. M. Nakhmanson,[1,3] S. Pamir Alpay,[1,3] and Jian-Xin Zhu[2,4]
[1)]Department of Materials Science & Engineering and Institute of Materials Science, University of Connecticut, Storrs, CT, 06269 - USA
[2)]Theoretical Division, Los Alamos National Laboratory, Los Alamos, NM, 87545 - USA
[3)]Department of Physics, University of Connecticut, Storrs, CT 06269 - USA
[4)]Center for Integrated Nanotechnologies, Los Alamos National Laboratory, Los Alamos, NM, 87545 - USA

SM-1: Demonstrative Figure to calculate average Distance between dopant atoms as performed for 25% doped supercells in Configuration I and Configuration II. (put the updated figure here)

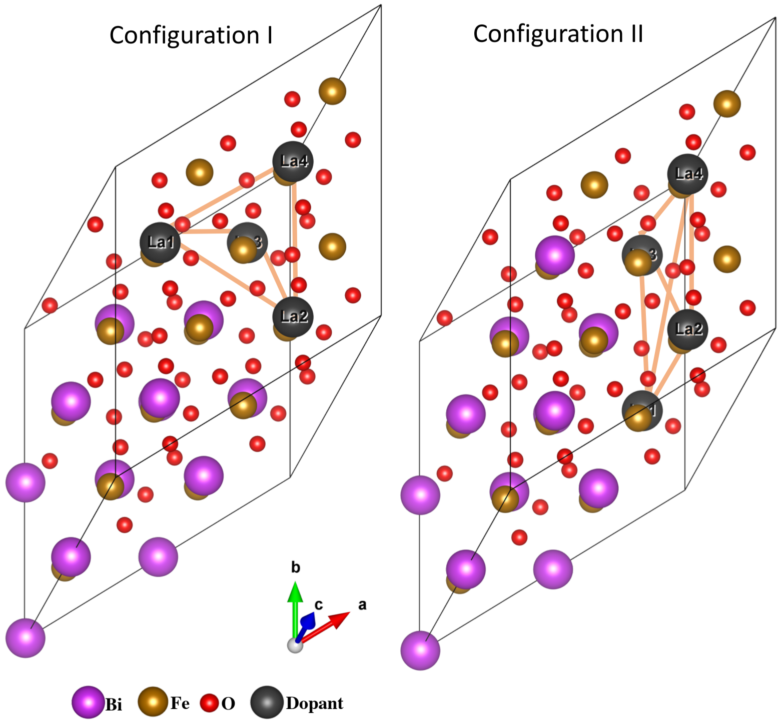

Configuration I (25% doped)	
Dopant atoms (La/Sr)	Distance(Å)
Dopant$_2$ –Dopant$_4$	5.69559
Dopant$_2$ –Dopant$_3$	5.56981
Dopant$_1$ –Dopant$_2$	5.56981
Dopant$_3$ –Dopant$_4$	5.69559
Dopant$_1$ –Dopant$_4$	5.69559
Dopant$_1$ –Dopant$_3$	5.56981

For Configuration I, the average distance between dopants = **8.44905 Å**

Configuration II (25% doped)	
Dopant atoms (La/Sr)	Distance(Å)
Dopant$_1$ –Dopant$_2$	5.69559
Dopant$_1$ –Dopant$_3$	5.69559
Dopant$_1$ –Dopant$_4$	9.93661
Dopant$_2$ –Dopant$_4$	5.69559
Dopant$_3$ –Dopant$_4$	5.69559
Dopant$_2$ –Dopant$_3$	5.56981

For Configuration II, the average distance between dopants = **9.572195 Å**

SM-2: Internal positions of A- and B-site atoms for 25% (a,b,c) and 50% (d,e,f) La (Sr) doped $BiFeO_3$ in the supercell for each configuration, measured in units of Bravais lattice vectors. *(For both percentages of dopants, Fe and O atoms positions remain fixed respectively in each configuration. Therefore, we report the positions of Fe and O atoms for only one configuration and all the A-site dopants in each case.)*

(a) 25 % Doped Configuration I								
Fe(x)	Fe(y)	Fe(z)	O(x)	O(y)	O(z)	Bi(x)	Bi(y)	Bi(z)
0.1134	0.1134	0.1134	0.2687	0.4730	0.1992	0.0028	0.0028	0.0028
0.1134	0.1134	0.6134	0.2687	0.4730	0.6992	0.0028	0.0028	0.5028
0.1134	0.6134	0.1134	0.2687	0.9730	0.1992	0.0028	0.5028	0.0028
0.1134	0.6134	0.6134	0.2687	0.9730	0.6992	0.0028	0.5028	0.5028
0.6134	0.1134	0.1134	0.7687	0.4730	0.1992	0.5028	0.0028	0.0028
0.6134	0.1134	0.6134	0.7687	0.4730	0.6992	0.5028	0.0028	0.5028
0.6134	0.6134	0.1134	0.7687	0.9730	0.1992	0.5028	0.5028	0.0028
0.6134	0.6134	0.6134	0.7687	0.9730	0.6992	0.5028	0.5028	0.5028
0.3634	0.3634	0.3634	0.1992	0.2687	0.4730	0.2528	0.2528	0.2528

0.3634	0.3634	0.8634	0.1992	0.2687	0.9730	0.2528	0.2528	0.7528
0.3634	0.8634	0.3634	0.1992	0.7687	0.4730	0.2528	0.7528	0.2528
0.3634	0.8634	0.8634	0.1992	0.7687	0.9730	0.7528	0.2528	0.2528
0.8634	0.3634	0.3634	0.6992	0.2687	0.4730	La/Sr(x)	La/Sr(y)	LaSr(z)
0.8634	0.3634	0.8634	0.6992	0.2687	0.9730	0.2528	0.7528	0.7528
0.8634	0.8634	0.3634	0.6992	0.7687	0.4730	0.7528	0.2528	0.7528
0.8634	0.8634	0.8634	0.6992	0.7687	0.9730	0.7528	0.7528	0.2528
			0.4730	0.1992	0.2687	0.7528	0.7528	0.7528
			0.4730	0.1992	0.7687			
			0.4730	0.6992	0.2687			
			0.4730	0.6992	0.7687			
			0.9730	0.1992	0.2687			
			0.9730	0.1992	0.7687			
			0.9730	0.6992	0.2687			
			0.9730	0.6992	0.7687			
			0.2230	0.0187	0.4492			
			0.2230	0.0187	0.9492			
			0.2230	0.5187	0.4492			
			0.2230	0.5187	0.9492			
			0.7230	0.0187	0.4492			
			0.7230	0.0187	0.9492			
			0.7230	0.5187	0.4492			
			0.7230	0.5187	0.9492			
			0.0187	0.4492	0.2230			
			0.0187	0.4492	0.7230			
			0.0187	0.9492	0.2230			
			0.0187	0.9492	0.7230			
			0.5187	0.4492	0.2230			
			0.5187	0.4492	0.7230			
			0.5187	0.9492	0.2230			
			0.5187	0.9492	0.7230			
			0.4492	0.2230	0.0187			
			0.4492	0.2230	0.5187			
			0.4492	0.7230	0.0187			
			0.4492	0.7230	0.5187			
			0.9492	0.2230	0.0187			
			0.9492	0.2230	0.5187			
			0.9492	0.7230	0.0187			
			0.9492	0.7230	0.5187			

(b) 25 % Doped Configuration II					
Bi(x)	Bi(y)	Bi(z)	La/Sr(x)	La/Sr(y)	La/Sr(z)
0.0028	0.0028	0.0028	0.7528	0.2528	0.2528
0.0028	0.0028	0.5028	0.7528	0.2528	0.7528
0.0028	0.5028	0.0028	0.7528	0.7528	0.2528
0.0028	0.5028	0.5028	0.7528	0.7528	0.7528
0.5028	0.0028	0.0028			
0.5028	0.0028	0.5028			
0.5028	0.5028	0.0028			
0.5028	0.5028	0.5028			
0.2528	0.2528	0.2528			
0.2528	0.2528	0.7528			
0.2528	0.7528	0.2528			
0.2528	0.7528	0.7528			

(c) 25 % Doped Configuration II					
Bi(x)	Bi(y)	Bi(z)	La/Sr(x)	La/Sr(y)	La/Sr(z)
0.0028	0.0028	0.5028	0.0028	0.0028	0.0028
0.0028	0.5028	0.0028	0.5028	0.5028	0.5028
0.0028	0.5028	0.5028	0.2528	0.2528	0.2528
0.5028	0.0028	0.0028	0.7528	0.7528	0.7528
0.5028	0.0028	0.5028			
0.5028	0.5028	0.0028			
0.2528	0.2528	0.7528			
0.2528	0.7528	0.2528			
0.2528	0.7528	0.7528			
0.7528	0.2528	0.2528			
0.7528	0.2528	0.7528			
0.7528	0.7528	0.2528			

(d) 50 % Doped Configuration I								
Fe(x)	Fe(y)	Fe(z)	O(x)	O(y)	O(z)	Bi(x)	Bi(y)	Bi(z)
0.1134	0.1134	0.1134	0.2687	0.4730	0.1992	0.0028	0.0028	0.5028
0.1134	0.1134	0.6134	0.2687	0.4730	0.6992	0.0028	0.5028	0.0028
0.1134	0.6134	0.1134	0.2687	0.9730	0.1992	0.0028	0.5028	0.5028
0.1134	0.6134	0.6134	0.2687	0.9730	0.6992	0.5028	0.0028	0.0028
0.6134	0.1134	0.1134	0.7687	0.4730	0.1992	0.5028	0.0028	0.5028

0.6134	0.1134	0.6134	0.7687	0.4730	0.6992	0.5028	0.5028	0.0028
0.6134	0.6134	0.1134	0.7687	0.9730	0.1992	0.5028	0.5028	0.5028
0.6134	0.6134	0.6134	0.7687	0.9730	0.6992	0.7528	0.7528	0.7528
0.3634	0.3634	0.3634	0.1992	0.2687	0.4730	La/Sr(x)	La/Sr(y)	La/Sr(z)
0.3634	0.3634	0.8634	0.1992	0.2687	0.9730	0.0028	0.0028	0.0028
0.3634	0.8634	0.3634	0.1992	0.7687	0.4730	0.2528	0.2528	0.2528
0.3634	0.8634	0.8634	0.1992	0.7687	0.9730	0.2528	0.2528	0.7528
0.8634	0.3634	0.3634	0.6992	0.2687	0.4730	0.2528	0.7528	0.2528
0.8634	0.3634	0.8634	0.6992	0.2687	0.9730	0.2528	0.7528	0.7528
0.8634	0.8634	0.3634	0.6992	0.7687	0.4730	0.7528	0.2528	0.2528
0.8634	0.8634	0.8634	0.6992	0.7687	0.9730	0.7528	0.2528	0.7528
			0.4730	0.1992	0.2687	0.7528	0.7528	0.2528
			0.4730	0.1992	0.7687			
			0.4730	0.6992	0.2687			
			0.4730	0.6992	0.7687			
			0.9730	0.1992	0.2687			
			0.9730	0.1992	0.7687			
			0.9730	0.6992	0.2687			
			0.9730	0.6992	0.7687			
			0.2230	0.0187	0.4492			
			0.2230	0.0187	0.9492			
			0.2230	0.5187	0.4492			
			0.2230	0.5187	0.9492			
			0.7230	0.0187	0.4492			
			0.7230	0.0187	0.9492			
			0.7230	0.5187	0.4492			
			0.7230	0.5187	0.9492			
			0.0187	0.4492	0.2230			
			0.0187	0.4492	0.7230			
			0.0187	0.9492	0.2230			
			0.0187	0.9492	0.7230			
			0.5187	0.4492	0.2230			
			0.5187	0.4492	0.7230			
			0.5187	0.9492	0.2230			
			0.5187	0.9492	0.7230			
			0.4492	0.2230	0.0187			
			0.4492	0.2230	0.5187			
			0.4492	0.7230	0.0187			
			0.4492	0.7230	0.5187			

	0.9492	0.2230	0.0187	
	0.9492	0.2230	0.5187	
	0.9492	0.7230	0.0187	
	0.9492	0.7230	0.5187	

(e) 50 % Doped Configuration II					
Bi(x)	Bi(y)	Bi(z)	La/Sr(x)	La/Sr(y)	La/Sr(z)
0.0028	0.0028	0.0028	0.2528	0.2528	0.2528
0.0028	0.0028	0.5028	0.2528	0.2528	0.7528
0.0028	0.5028	0.0028	0.2528	0.7528	0.2528
0.0028	0.5028	0.5028	0.2528	0.7528	0.7528
0.5028	0.0028	0.0028	0.7528	0.2528	0.2528
0.5028	0.0028	0.5028	0.7528	0.2528	0.7528
0.5028	0.5028	0.0028	0.7528	0.7528	0.2528
0.5028	0.5028	0.5028	0.7528	0.7528	0.7528

(f) 50 % Doped Configuration III					
Bi(x)	Bi(y)	Bi(z)	La/Sr(x)	La/Sr(y)	La/Sr(z)
0.0028	0.0028	0.0028	0.5028	0.5028	0.5028
0.0028	0.0028	0.5028	0.2528	0.2528	0.7528
0.0028	0.5028	0.0028	0.2528	0.7528	0.2528
0.0028	0.5028	0.5028	0.2528	0.7528	0.7528
0.5028	0.0028	0.0028	0.7528	0.2528	0.2528
0.5028	0.0028	0.5028	0.7528	0.2528	0.7528
0.5028	0.5028	0.0028	0.7528	0.7528	0.2528
0.2528	0.2528	0.2528	0.7528	0.7528	0.7528

SM-3: Comparison of total energies for all undoped and doped systems in the primitive unit cell and the supercell with G-type AFM and FM ordering.

System	G-type AFM/FM	Total energy (eV)
Unit cell		
BFO pristine	G-type AFM	-60.10035965
BFO pristine	FM	-59.47373816
$BiLaFe_2O_6$	G-type AFM	-71.91013520
$BiLaFe_2O_6$	FM	-70.95266660
$BiSrFe_2O6$	G-type AFM	-65.38731136
$BiSrFe_2O6$	FM	-64.31028036
$LaFeO_3$	G-type AFM	-77.67134534
$LaFeO_3$	FM	-77.01842113

$SrFeO_3$	G-type AFM	-64.53768348
$SrFeO_3$	FM	-64.21991046
Supercell		
BFO pristine	G-type AFM	-520.69212000
BFO pristine	FM	-517.72300359
6% La Doped BFO	G-type AFM	-527.21689614
6% La Doped BFO	FM	-524.24699924
6% Sr Doped BFO	G-type AFM	-521.08715479
6% Sr Doped BFO	FM	-518.49603827
25% La doped BFO – Configuration I	G-type AFM	-546.95615885
25% La doped BFO – Configuration I	FM	-544.03175768
25% La doped BFO – Configuration II	G-type AFM	-546.97171173
25% La doped BFO – Configuration II	FM	-544.04871712
25% La doped BFO – Configuration III	G-type AFM	-546.90667696
25% La doped BFO – Configuration III	FM	-543.97586602
25% Sr doped BFO – Configuration I	G-type AFM	-521.91935454
25% Sr doped BFO – Configuration I	FM	-520.53044359
25% Sr doped BFO – Configuration II	G-type AFM	-521.91251341
25% Sr doped BFO – Configuration II	FM	-520.54317248
25% Sr doped BFO – Configuration III	G-type AFM	-521.94937117
25% Sr doped BFO – Configuration III	FM	-520.58215380
50% La doped BFO – Configuration I	G-type AFM	-573.24698963
50% La doped BFO – Configuration I	FM	-569.94256727
50% La doped BFO – Configuration II	G-type AFM	-573.28540190
50% La doped BFO – Configuration II	FM	-570.27155100
50% La doped BFO – Configuration III	G-type AFM	-573.24695683
50% La doped BFO – Configuration III	FM	-569.89000329
50% Sr doped BFO – Configuration I	G-type AFM	-522.82385584
50% Sr doped BFO – Configuration I	FM	-522.23664707
50% Sr doped BFO – Configuration II	G-type AFM	-523.15006508
50% Sr doped BFO – Configuration II	FM	-522.59557407
50% Sr doped BFO – Configuration III	G-type AFM	-522.82385503
50% Sr doped BFO – Configuration III	FM	-522.23664414

SM-4: Local magnetic moment on Fe atom and its dependence on the distance (in ascending order) of Fe sites away from the dopant atoms in 6% doped case.

Distance (Å)	Fe Moments (μ_B) in $La_{0.06}Bi_{0.94}FeO_3$	Fe Moments (μ_B) in $Sr_{0.06}Bi_{0.94}FeO_3$
3.117	-4.039	-3.963
3.3019	-4.04	-3.966
3.3019	4.039	3.963
3.3019	4.038	3.991
3.5771	-4.045	-4.024
3.5771	-4.044	-4.003
3.5771	4.045	3.969
3.9237	4.044	4.023
6.3375	-4.045	-4.014
6.3375	-4.045	-4.015
6.3375	4.045	4.026
7.0448	-4.046	-4.025
7.0448	4.046	4.002
7.0448	4.046	4.01
10.1577	-4.044	-4.023
10.9643	4.046	4.01

SM-5: Bader charges and volumes on Bi, Fe and O atoms for pristine $BiFeO_3$ supercell. The total number of valence electrons for each element as considered in each pseudpotential are also listed here in parenthesis for reference.

Pristine BFO supercell					
Bi (15)		Fe (8)		O (6)	
Charge	Volume(Å^3)	Charge	Volume(Å^3)	Charge	Volume(Å^3)
12.320901	13.407537	6.233552	7.277843	7.493266	14.087122
12.320899	13.407537	6.233555	7.277843	7.493263	14.087122
12.320900	13.407537	6.233552	7.277843	7.493266	14.087122
12.320900	13.407537	6.233554	7.277843	7.493264	14.087122
12.320899	13.407537	6.233553	7.277843	7.493266	14.087122
12.320900	13.407537	6.233555	7.277843	7.493265	14.087122
12.320899	13.407537	6.233553	7.277843	7.493267	14.087122
12.320901	13.407537	6.233554	7.277843	7.493266	14.087122
12.320008	13.405619	6.234200	7.280720	7.470968	14.034846
12.320009	13.405619	6.234204	7.280720	7.470969	14.034846
12.320010	13.405619	6.234201	7.280720	7.470968	14.034846
12.320007	13.405619	6.234202	7.280720	7.470969	14.034846
12.320008	13.405619	6.234283	7.281200	7.470968	14.034846
12.320007	13.405619	6.234202	7.280720	7.470969	14.034846

12.320008	13.405619	6.234199	7.280720	7.470968	14.034846
12.320008	13.405619	6.234202	7.280720	7.470969	14.034846
				7.491536	14.077051
				7.491534	14.077051
				7.491535	14.077051
				7.491534	14.077051
				7.491535	14.077051
				7.491534	14.077051
				7.491533	14.077051
				7.491534	14.077051
				7.472374	14.037244
				7.472375	14.037244
				7.472374	14.037244
				7.472374	14.037244
				7.472374	14.037244
				7.472289	14.036765
				7.472375	14.037244
				7.472374	14.037244
				7.491236	14.080888
				7.491234	14.080888
				7.491236	14.080888
				7.491235	14.080888
				7.491236	14.080888
				7.491234	14.080888
				7.491236	14.080888
				7.491235	14.080888
				7.471956	14.033887
				7.471954	14.033887
				7.471955	14.033887
				7.471954	14.033887
				7.471955	14.033887
				7.471955	14.033887
				7.471955	14.033887
				7.471953	14.033887

SM-6: Bader charges and volumes on La and Sr dopant atoms for each doping concentration in the doped $BiFeO_3$ supercell. The total number of valence electrons for each element as considered in each pseudpotential are also listed here in parenthesis for reference.

6% doped			
La (11)		Sr (10)	
Charge	Volume(Å^3)	Charge	Volume(Å^3)
8.924499	17.960394	8.426785	15.363866
	25% doped		
8.922799	18.022512	8.425587	15.415813
8.922781	18.022512	8.425574	15.415813
8.923762	18.027200	8.425512	15.413682
8.923754	18.026987	8.425512	15.413682
	50% doped		
8.941205	18.019316	8.428153	15.494869
8.914977	17.982452	8.422097	15.358919
8.919349	17.984157	8.422101	15.377031
8.919506	17.983730	8.422393	15.377457
8.920017	17.990549	8.423956	15.385129
8.919466	17.986501	8.422374	15.378949
8.920385	17.994598	8.423729	15.381506
8.920553	17.994172	8.423688	15.380228

SM-7: Bader charges and volumes on Bi, Fe and O atoms for each doping concentration in the doped $BiFeO_3$ supercell. The total number of valence electrons for each element as considered in each pseudpotential are also listed here in parenthesis for reference.

6% La-doped					
Bi (15)		Fe (8)		O (6)	
Charge	Volume(Å^3)	Charge	Volume(Å^3)	Charge	Volume(Å^3)
12.153417	12.583221	6.169523	7.140928	7.588957	14.420793
12.152507	12.578979	6.169394	7.128837	7.581626	14.129980
12.149807	12.567100	6.169761	7.130534	7.588545	14.418672
12.152345	12.571767	6.165853	7.133504	7.588491	14.418460
12.149228	12.563070	6.160003	7.132019	7.589629	14.391521
12.150949	12.563706	6.165693	7.132868	7.588572	14.417823
12.153216	12.574100	6.165735	7.133292	7.586445	14.408490
12.151400	12.573464	6.165819	7.132231	7.588441	14.418884
12.155545	12.590221	6.167174	7.137110	7.564187	14.342946
12.153530	12.576433	6.167192	7.138171	7.564136	14.339976
12.153763	12.578130	6.166911	7.137534	7.567993	14.328522
12.156129	12.589372	6.169069	7.136049	7.564061	14.342522
				7.558560	14.063163
				7.564646	14.342946

12.152752	12.573252	6.167088	7.137958	7.565227	14.344643
12.155745	12.589160	6.169577	7.138171	7.564186	14.341249
12.150817	12.572615	6.169696	7.139231	7.587661	14.415066
		6.169159	7.140080	7.590633	14.394703
				7.582265	14.132314
				7.587281	14.409551
				7.587018	14.411036
				7.587509	14.413369
				7.586666	14.406581
				7.587314	14.411672
				7.566406	14.345704
				7.566388	14.351219
				7.570797	14.372430
				7.566868	14.347613
				7.567199	14.352491
				7.566333	14.349734
				7.297010	12.859186
				7.567921	14.358218
				7.589499	14.419732
				7.588841	14.416763
				7.587919	14.414642
				7.587685	14.413793
				7.591394	14.434369
				7.314884	12.901397
				7.590868	14.427369
				7.590492	14.426308
				7.567025	14.348673
				7.571469	14.376673
				7.567378	14.351855
				7.297625	12.858125
				7.567736	14.355673
				7.567080	14.348461
				7.567438	14.354825
				7.568389	14.358431
6% Sr-doped					
Bi		Fe		O	
Charge	Volume(Å^3)	Charge	Volume(Å^3)	Charge	Volume(Å^3)
12.151139	12.576433	6.117826	7.163625	7.583267	14.403824
12.153868	12.584070	6.118440	7.168928	7.551995	14.553366
12.150643	12.568161	6.118726	7.159170	7.565755	14.341461
12.148939	12.545676	6.117888	7.141989	7.583735	14.407218
12.148600	12.556282	6.118652	7.160019	7.570764	14.497155
12.149197	12.546313	6.116886	7.137534	7.576380	14.383036
12.151710	12.559888	6.116753	7.137110	7.580620	14.382612
12.150975	12.571342	6.117077	7.137958	7.584876	14.409551
12.149005	12.565615	6.119008	7.147928	7.562866	14.341249
12.147617	12.559888	6.118072	7.142837	7.553158	14.298401
12.152787	12.611221	6.118191	7.144110	7.556472	14.454944

12.147486	12.560737	6.118097	7.153443	7.562214	14.329795
12.151195	12.603372	6.118197	7.144958	7.554031	14.560790
12.152874	12.613130	6.117989	7.148776	7.562263	14.327886
12.150738	12.571342	6.118131	7.149837	7.552190	14.306674
		6.117371	7.169776	7.561713	14.334037
				7.586016	14.409763
				7.577954	14.518155
				7.559750	14.580093
				7.570433	14.355461
				7.571683	14.357370
				7.579949	14.380915
				7.585564	14.406793
				7.580459	14.391097
				7.560044	14.321522
				7.561819	14.333401
				7.566014	14.537033
				7.561462	14.334886
				7.564860	14.344643
				7.563907	14.346764
				7.269357	13.211088
				7.561369	14.333401
				7.586242	14.406157
				7.581924	14.395551
				7.585733	14.407005
				7.582155	14.395339
				7.583439	14.585820
				7.258133	13.184149
				7.588090	14.423763
				7.582998	14.397460
				7.554649	14.305189
				7.566274	14.536609
				7.562219	14.333401
				7.266100	13.202816
				7.562201	14.333825
				7.562683	14.340188
				7.562151	14.336582
				7.562827	14.338492
25% La-doped					
Bi		Fe		O	
Charge	Volume(Å^3)	Charge	Volume(Å^3)	Charge	Volume(Å^3)
12.161942	12.656970	6.166248	7.182113	7.313856	12.960619
12.162173	12.658461	6.164609	7.171672	7.580700	14.190347
12.156977	12.628416	6.164000	7.169328	7.589152	14.494849

12.156977	12.628416	6.164069	7.168902	7.592807	14.472049
12.162173	12.658461	6.164069	7.168902	7.592808	14.472049
12.161943	12.656970	6.164000	7.169328	7.589152	14.494849
12.160069	12.647168	6.164534	7.171459	7.580698	14.190347
12.162587	12.658461	6.166248	7.182113	7.313856	12.960619
12.156573	12.626711	6.164485	7.173590	7.294836	12.912888
12.156572	12.626711	6.164609	7.163788	7.567994	14.426235
12.162588	12.658461	6.163612	7.167623	7.565835	14.379356
12.160069	12.647168	6.163698	7.167623	7.558608	14.126634
		6.163699	7.167623	7.558609	14.126634
		6.163611	7.167623	7.565835	14.379356
		6.162618	7.163788	7.567994	14.426235
		6.164485	7.173590	7.294836	12.912888
				7.313087	12.956571
				7.588496	14.447331
				7.580086	14.188642
				7.589159	14.492079
				7.589159	14.492079
				7.580010	14.188429
				7.588496	14.447331
				7.313087	12.956571
				7.560473	14.132814
				7.295295	12.912462
				7.568804	14.428792
				7.568565	14.390436
				7.568565	14.390436
				7.568805	14.428792
				7.295296	12.912462
				7.560481	14.133027
				7.580784	14.190773
				7.593170	14.472049
				7.313139	12.956571
				7.589732	14.496341
				7.589733	14.496341
				7.313140	12.956571
				7.593170	14.472049
				7.580784	14.190773
				7.561146	14.135797
				7.568989	14.430923
				7.568673	14.389584
				7.296469	12.916723
				7.296468	12.916723
				7.568673	14.389584
				7.568989	14.430923

				7.561145	14.135797
25% Sr-doped					
Bi		Fe		O	
Charge	Volume(Å^3)	Charge	Volume(Å^3)	Charge	Volume(Å^3)
12.148735	12.631612	6.122824	7.248810	7.243177	13.197786
12.153122	12.654839	6.123231	7.220682	7.528692	14.533205
12.146764	12.623089	6.122910	7.223665	7.542113	14.520633
12.146764	12.623089	6.122132	7.222600	7.560046	14.541089
12.153122	12.654839	6.122132	7.222600	7.560046	14.541089
12.148735	12.631612	6.122910	7.223665	7.542113	14.520633
12.145263	12.616270	6.123231	7.220682	7.528691	14.533205
12.152243	12.651216	6.122824	7.248810	7.243177	13.197786
12.146466	12.620745	6.122459	7.250301	7.254248	13.224422
12.146466	12.620745	6.121294	7.217912	7.539285	14.511044
12.152243	12.651216	6.122716	7.217060	7.545765	14.480359
12.145263	12.616270	6.122446	7.216207	7.528298	14.525321
		6.122446	7.216207	7.528299	14.525321
		6.122717	7.217060	7.545765	14.480359
		6.121295	7.217912	7.539285	14.511044
		6.122459	7.250301	7.254248	13.224422
				7.250392	13.216324
				7.564927	14.551317
				7.532321	14.543007
				7.546725	14.533418
				7.546725	14.533418
				7.532320	14.543007
				7.564927	14.551317
				7.250392	13.216324
				7.532883	14.541728
				7.255834	13.230601
				7.538557	14.503799
				7.546777	14.479081
				7.546777	14.479081
				7.538557	14.503799
				7.255835	13.230601
				7.532883	14.541728
				7.535553	14.554514
				7.565114	14.556005
				7.251813	13.219308
				7.547305	14.531500
				7.547305	14.531500
				7.251814	13.219308
				7.565114	14.556005

				7.535553	14.554514
				7.527800	14.523403
				7.537155	14.506356
				7.548987	14.501668
				7.252530	13.220799
				7.252529	13.220799
				7.548987	14.501668
				7.537155	14.506356
				7.527800	14.523403
50% La-doped					
Bi		Fe		O	
Charge	Volume(Å^3)	Charge	Volume(Å^3)	Charge	Volume(Å^3)
12.169405	12.669542	6.165592	7.176786	7.320527	12.981076
12.168953	12.668903	6.164902	7.178491	7.322506	12.996205
12.168131	12.674017	6.164888	7.176573	7.321925	12.986829
12.169496	12.671886	6.158242	7.175721	7.325487	12.980650
12.170800	12.687654	6.164864	7.175934	7.321088	12.991943
12.171324	12.690424	6.158714	7.177638	7.314771	12.953374
12.176786	12.709389	6.158327	7.174016	7.316010	12.713864
12.148975	12.594322	6.158358	7.160378	7.591309	14.493571
		6.157515	7.164427	7.301963	12.933131
		6.157566	7.161870	7.303286	12.937180
		6.157643	7.162722	7.302111	12.942294
		6.159316	7.165066	7.295958	12.663789
		6.157591	7.163362	7.302868	12.943999
		6.158551	7.163575	7.306554	12.931000
		6.158890	7.163148	7.295425	12.901807
		6.168535	7.180195	7.570007	14.430710
				7.320117	12.980437
				7.320399	12.991730
				7.320929	12.991091
				7.313961	12.950391
				7.321098	12.984485
				7.314720	12.708537
				7.324363	12.976175
				7.591555	14.495702
				7.563352	14.111931
				7.293288	12.620745
				7.561658	14.105965
				7.563584	14.113849
				7.557517	14.118537
				7.566525	14.136862
				7.561193	14.101064
				7.566889	14.378503

				7.583807	14.169465
				7.581716	14.195248
				7.311357	12.662510
				7.586419	14.189708
				7.580824	14.154335
				7.581314	14.156679
				7.584091	14.168825
				7.587175	14.440938
				7.562351	14.108948
				7.561124	14.102555
				7.558309	14.121307
				7.561297	14.104047
				7.294078	12.623089
				7.562202	14.108522
				7.565799	14.131535
				7.566362	14.375733
50% Sr-doped					
Bi		Fe		O	
Charge	Volume(Å^3)	Charge	Volume(Å^3)	Charge	Volume(Å^3)
12.147437	12.671247	6.129627	7.331061	7.215268	13.291544
12.148979	12.678278	6.129099	7.312949	7.227904	13.337358
12.146510	12.708111	6.132984	7.319129	7.207733	13.261925
12.144876	12.656117	6.127588	7.277150	7.211279	13.412791
12.146125	12.704488	6.130352	7.315506	7.228591	13.328195
12.145643	12.702997	6.126654	7.270971	7.215556	13.112338
12.153652	12.763940	6.127292	7.276085	7.174639	13.402350
12.135852	12.499924	6.128476	7.275872	7.554091	14.559202
		6.124776	7.276937	7.231428	13.325212
		6.122607	7.276085	7.227050	13.304756
		6.124565	7.278855	7.237494	13.346095
		6.128858	7.292493	7.195453	13.449229
		6.125786	7.282477	7.239840	13.362929
		6.128342	7.293345	7.223959	13.437722
		6.128992	7.298672	7.230842	13.143022
		6.123164	7.255202	7.541224	14.507848
				7.221324	13.310083
				7.229134	13.334588
				7.228751	13.341833
				7.214132	13.111272
				7.210723	13.276202
				7.176432	13.410234
				7.211456	13.417053
				7.549054	14.535762
				7.509445	14.602032

				7.187247	13.369534
				7.515968	14.623128
				7.504077	14.570282
				7.511964	14.464804
				7.519166	14.824495
				7.503942	14.562611
				7.521434	14.374455
				7.491651	14.557497
				7.499020	14.434332
				7.165800	13.322229
				7.504873	14.784648
				7.507928	14.607359
				7.493277	14.541089
				7.488463	14.534483
				7.520144	14.379356
				7.508681	14.603737
				7.513310	14.617161
				7.510811	14.459903
				7.502822	14.563463
				7.183921	13.363568
				7.501511	14.566873
				7.513664	14.807448
				7.520788	14.376373
